# Biochemical predictors for Sars-Cov-2 severity

**DOI:** 10.6026/97320630017834

**Published:** 2021-09-30

**Authors:** Ezhil Gurusamy, S Mahalakshmi, Gurumoorthy Kaarthikeyan, K Ramadevi, Parkavi Arumugam, MS Gayathri

**Affiliations:** 1 Institute of Biochemistry, Madras Medical College, RGGGH, Chennai, India; 2 Saveetha Dental College & Hospital, Saveetha Institute of Medical and Technical Sciences (SIMATS), Chennai - 77,India; 3 Institute of Biochemistry, Stanley Medical College, Chennai, India

**Keywords:** Biochemical, predictor, Sars-Cov-2, severity

## Abstract

It is of interest to assess the inflammatory marker profile in SARS-CoV-2 patients and to correlate the levels of systemic inflammatory biomarkers such as neutrophil-to-lymphocyte ratio (NLR), C-Reactive Protein CRP, Ferritin, Creatine kinase (CK), Lactate dehydrogenase (LDH) and liver function analytes total serum proteins, albumin, total bilirubin, direct bilirubin, alkaline phosphatase, aspartate aminotransferase (AST) and alanine aminotransferase (ALT) with the severity of SARS-CoV-2 infections. A total of 1000 COVID-19 positive patient's data were collected. Laboratory assessments consisted of NLR (neutrophil-lymphocyte ratio) by cell counter, C Reactive Protein (CRP) by immunoturbidimetry, Ferritin by electrochemiluminescence (ECLIA) and Creatine Kinase (CK), Lactate Dehydrogenase (LDH), Alkaline phosphatase (ALP), Aspartate aminotransferase (AST), Alanine aminotransferase (ALT), Total Bilirubin, Direct Bilirubin, Total Protein and Albumin by spectrophotometry. The mean plasma CRP levels, NLR, ferritin, CK and LDH levels were higher in severe cases than in non-severe cases, and the difference was statistically significant (p<0.05). All liver function tests such as the total and direct bilirubin, AST, ALT, ALP, total protein and albumin were higher in severe patients than non-severe patients and the difference was statistically significant (p<0.05). Data indicate that NLR, CRP, Ferritin, CK, LDH and liver function analytes have a crucial role as prognostic markers for SARS-CoV-2 infections and hence should be routinely recommended for risk assessment and stratification of the patients to reduce the associated morbidity and mortality.

## Background:

The COVID-19 pandemic has led to dramatic loss of lives worldwide with unprecedented burden on the medical fraternity. It has displayed a broad spectrum of clinical symptoms and varied susceptibility across ages and populations. As soon as patients progress to the severity or critical stage, the risk for poor outcomes increases significantly [1,2]. It is estimated that around 10–15% of mild COVID-19 patients advance to severe disease and 15–20% of severe cases progress to become critical, with many of the individuals in the critical category needing treatment in intensive care units (ICU)[1,3]. Hence, it becomes imperative that severity predictive biomarkers be identified for early stratification, triage and efficient utilisation of resources in the treatment of individuals with SARS-CoV-2 infections.

The portal of entry of the virus into the host cell is through the angiotensin-converting enzyme-2 (ACE-2) receptors expressed by various cells [4]. On binding of the viral spike protein to the ACE-2 receptors on the host cell surface, conformational changes occur in the spike protein, which leads to fusion of the viral envelope with the host cell membrane [5,6]. Also, the virus is identified by pathogen recognition receptors like Toll like receptors (TLR), the nucleotide-binding oligomerization domain (NOD)- Leucin Rich Repeats (LRR)-containing receptors (NLR), the retinoic acid-inducible gene 1 (RIG-1) -like receptors (RLR) and the C-type lectin receptors (CLR) which detects the microbe-associated molecular patterns and activates adapter proteins such as Myeloid differentiation primary response 88 (MyD88), Toll-interleukin 1 receptor (TIR) domain-containing adapter protein (TIRAP), and TRIF-related adaptor molecule (TRAM)[7,8]. These proteins further activate Nuclear Factor kappa-light-chain-enhancer of activated B cells (NF-kB) and Mitogen-activated protein kinases (MAPK) signalling pathways which stimulate cellular responses and release of inflammatory mediators like interleukins, interferons, Tumour necrosis factor-A, C-reactive proteins [9]. The cytokines and chemokines thus released cause a cytokine storm with associated systemic inflammation, increased risk of Acute Respiratory Distress Syndrome, Disseminated Intravascular Coagulation leading to Multiple Organ Dysfunction Syndrome [10].

Peiris et al. in 2003 conducted a study to analyse the clinical progression and viral load in a community outbreak of coronavirus-associated Severe Acute Respiratory Syndrome (SARS) pneumonia and found it to have a triphasic pattern [11]. Analysing the available evidence, it may be postulated that SARS-CoV-2 infections may also express a similar phasic pattern of disease progression. Various studies have suggested that higher levels of inflammatory markers such as WBC, CRP, PCT, ESR, IL-6, and IL-10 are associated with the severity of COVID-19[1,12-16]. Therefore, It is of interest to assess the inflammatory marker profile in SARS-CoV-2 patients and to correlate the levels of systemic inflammatory biomarkers such as neutrophil-to-lymphocyte ratio (NLR), CRP, Ferritin, Creatine kinase (CK), Lactate dehydrogenase (LDH) and liver function analytes total serum proteins, albumin, total bilirubin, direct bilirubin, alkaline phosphatase, aspartate aminotransferase (AST) and alanine aminotransferase (ALT) with the severity of SARS-CoV-2 infections.

## Materials & Methods:

This was a retrospective, single-centered cohort study conducted at the Rajiv Gandhi Government General Hospital, Chennai which is the designated hospital for the treatment of COVID-19 patients. Data of patients were collected from June 2020 to August 2020 from the Institute of Biochemistry and Pathology, Rajiv Gandhi Government General Hospital. Ethical clearance was obtained from the institutional ethical committee before the start of the study. A total of 1000 COVID-19 positive patient's data were collected from June 2020 to August 2020, of which (35%) 350 were female patients and (65%) 650 were male patients.

Only laboratory-confirmed COVID-19 patients with mild symptoms at the time of admission were enrolled in this study. A confirmed case of COVID-19 was defined as a positive result on real-time Reverse-Transcriptase-Polymerase-Chain-Reaction (RT-PCR) assay using nasal or pharyngeal swab specimens. Laboratory assessments consisted of NLR (Neutrophil-Lymphocyte Ratio) by cell counter, C Reactive Protein (CRP) by immunoturbidimetry, Ferritin by electrochemiluminescence (ECLIA) and Creatine Kinase (CK), Lactate Dehydrogenase (LDH), Alkaline phosphatase (ALP), Aspartate aminotransferase (AST), Alanine aminotransferase (ALT), Total Bilirubin, Direct Bilirubin, Total Protein and Albumin by spectrophotometry. Demographic information, clinical signs and symptoms, clinical outcomes and laboratory findings on admission were extracted from the electronic medical records. Only the baseline laboratory data at the time of admission before undergoing treatment were collected regardless of cases receiving multiple laboratory testing.

In the present study only, the inclusion criteria were patients confirmed with SARS-CoV-2 infection by real-time reverse transcription polymerase chain reaction (RT-PCR), COVID-19 positive patients with presence of lung involvement confirmed by CT-CHEST findings and COVID-19 positive patients of all age groups. COVID-19 positive patients for whom the treatment had already been started and COVID-19 positive patients without lung involvement were excluded from the study. Non-probability convenience sampling technique was used. Chi square association test was used to analyse the associations.

## Results:

The mean age of the study participants was 52.6 ± 15.8 years. The most common age group affected was 50-75 years, which can be attributed to the associated comorbidities in this age group. The female participants were significantly younger (mean age: 48.4 ± 16.4 years) than the male participants (mean age: 54.6 ± 15.2 years).

The mean plasma CRP levels, NLR, ferritin, CK and LDH levels were higher in severe cases than in non-severe cases, and the difference was statistically significant (p<0.05). All liver function test analytes - Total and direct bilirubin, AST, ALT, ALP, total protein and albumin were higher in severe patients than non-severe patients and the difference was statistically significant (p<0.05).

To identify the association of NLR with CRP levels and ferritin levels in severe and non-severe patients, a correlation analysis was conducted. In severe patients, the results showed that NLR was positively correlated with both CRP levels (R=0.0001, FIG-1) and ferritin (R=-0.0001, FIG-2). Plasma CRP levels also positively correlated with ferritin levels (R=0.0001, FIG-3). Similarly, LDH levels positively correlated with NLR (R=0.001), CRP (R=0.001), Ferritin (R=0.001) and CK levels (R=0.001). CK levels positively correlated with CRP (R=0.001), ferritin (R=0.001) and LDH levels (R=0.001) but negatively correlated with NLR (R=0.218).

On comparison of all liver function analytes with the inflammatory markers, it was observed that direct bilirubin, AST, ALT and albumin had significant positive correlation with the inflammatory markers. Total bilirubin had positive correlation with all inflammatory markers except NLR. ALP had positive correlation only with CRP and CK while it had a negative correlation with NLR, ferritin and LDH. Total protein had a positive correlation with NLR, CK and Ferritin while it had a negative correlation with CRP and LDH levels.

## Discussion:

Managing COVID-19 infections worldwide has become a huge task for the medical fraternity. The rapid progression of the disease from being mild symptomatic to severe breathlessness with reduced oxygen saturation and with lack of adequate medical infrastructure it becomes paramount that early symptom profiling and stratification based on prognostic biomarkers be done for all patients to reduce the associated morbidity and mortality. The present study was conducted to analyse and correlate the biochemical inflammatory markers like NLR, CRP, Ferritin with the clinical symptoms of the patients. CK, LDH and liver function analytes were assessed and correlated with the inflammatory markers, which was predictive of multi-organ involvement.

Neutrophil-to-lymphocyte ratio (NLR) is the most well established inflammatory marker that reflects systemic inflammatory response and is easily obtainable through routine blood count analysis [1,17]. In the present study, high NLR levels correlated with severity of COVID-19 infections reflecting the enhanced inflammatory process in severe/critical COVID-19 patients. These findings are supported by recent meta-analysis by Roshan Kumar Mahat et al and Pan Ji et al where NLR values were significantly associated with the severity and mortality of COVID-19 infections [12]. Neutrophil (NEU) is a major component of the leukocyte population and can kill pathogens by releasing reactive oxygen species, producing effector molecules such as circulating vascular endothelial growth factor (VEGF), and inducing inflammatory factors as well as IL1, TNF-α, and IFN-γ [18,19]. Thus, because of the human immune response and cytokines produced by lymphocyte and endothelial cells, elevated NLR values may be seen following COVID-19 infections. Therefore, NLR levels can be used as functional prognostic markers for severity of COVID-19 infections.

C-reactive protein is an acute-phase inflammatory protein produced by the liver and regulated at the transcriptional level by the cytokine IL-6 and IL-1 [20]. It is an important index for diagnosing and evaluating severe pulmonary infectious diseases [1,21]. In this study, CRP levels correlated with severity of COVID-19 infections. These findings are supported by recent meta-analysis by Roshan Kumar Mahat et al. and Pan Ji et al. [1,12]. It is also noteworthy that our results are also in keeping with those of previous studies [12, [22,23,24]. The National Health Commission of the People's Republic of China included elevated inflammatory factors such as IL-6 and CRP as potential early warning indicators of severe disease in its widely used "COVID-19 diagnosis and treatment plan"[12,25]. As the first line of innate host defences for clearance of viral infections, CRP might be linked to the overproduction of inflammatory cytokines in severe patients and may lead to dysfunction of various organ systems in COVID-19-infected patients [18,26, 27]. A positive correlation between CRP levels and lung lesions, kidney damage, and cardiac injury has been demonstrated; when the inflammation or tissue damage is resolved, CRP concentration falls [18, 28,29]. Hence, it can be concluded that CRP levels have a major role as an early prognostic marker for severity of COVID-19 infections.

Ferritin, an important acute phase reactant, helps in host modulation by restricting the availability of iron to pathogens. It also regulates cytokine synthesis and release. Along with significant activation of macrophages, patients manifesting hyperferritinemic phenotype show an abnormal pattern of activation of reticuloendothelial system and multiple organ damage [7,30]. Ferritin levels correlated with the severity of COVID-19 infections. This is in accordance with the meta-analysis by Roshan kumar Mahat et al. [1], which states that higher serum ferritin level was associated with mortality in COVID-19 patients. Though the pathophysiological background responsible for the association of hyperferritinemia and disease severity in patients with COVID-19 is not clearly grasped, it is suggested that hyperferritinemia in COVID-19 patients is most likely due to the cytokine storm and a secondary hemophagocytic lymphohistiocytosis [1,31]. Along with it having value as an early prognostic marker it also has a role as target for therapeutic interventions.

In the present study CK correlated with all inflammatory markers except NLR while LDH significantly correlated with all inflammatory markers. The increase in CK and LDH levels are suggestive of myocardial tissue damage and can be used as early prognostic markers to assess myocardial involvement in COVID-19 patients. Similarly, all liver function analytes significantly correlated with the inflammatory markers suggesting early liver involvement and damage in severe COVID-19 infections except ALP and total proteins. The direct bilirubin was abnormally high in nearly 45% of patients, but total bilirubin was abnormal only in 7.9%. But the Total and direct bilirubin levels significantly correlated with all inflammatory markers except NLR. Nearly 49.5% of the study population had hypoproteinemia, which significantly correlated with ferritin and NLR levels in severe infections. 70% of covid positive patients have abnormal Albumin levels, which highly significantly correlated with Ferritin, CRP & NLR levels in severe infections. These findings were in accordance with studies by Muhammad Sohaib Asghar et al, Abeer Altaf et al, Yafei Zhang et al and meta analysis by Sulmaz Ghahramani et al. [32-35]. This evidence highlights the importance of monitoring the liver function analytes level to determine the progression of the infection and prevent multi-organ involvement. It also suggests that ALP and total protein levels might not have greater significance as a prognostic biomarker.

## Conclusion:

Data (Figures 1 to 3 and Tables 1 to 5 - see PDF) shows that NLR, CRP, Ferritin, CK, LDH and liver function analytes have a crucial role as prognostic markers for SARS-CoV-2 infections. This should be routinely recommended for risk assessment and stratification of the patients to reduce the associated morbidity and mortality. Similar data is needed among various other populations at a larger scale to provide more insight regarding the role of biochemical markers in predicting the course of the disease.
